# Energy, technology, and economic growth in Saudi Arabia: An ARDL and VECM analysis approach

**DOI:** 10.1016/j.heliyon.2024.e26033

**Published:** 2024-02-08

**Authors:** Faten Derouez, Adel Ifa, Abdullah A. Aljughaiman, Mohammed Bu Haya, Abdalwali Lutfi, Mahmaod Alrawad, Samah Bayomei

**Affiliations:** a Quantitative Methods Department, School of Business, King Faisal University, Kingdom of Saudi Arabia; b University of Sousse, Higher Institute of Finance and Taxation, Sousse, Tunisia; c Finance Department, School of Business, King Faisal University, Kingdom of Saudi Arabia; d College of Business Administration, The University of Kalba, Kalba, 11115, UAE; e Accounting Department, School of Business, King Faisal University, Kingdom of Saudi Arabia; f College of Business Administration and Economics, Al-Hussein Bin Talal University, Ma'an, 71111, Jordan; g Applied Science Research Center, Applied Science Private University, Jordan; h MEU Research Unit, Middle East University, Amman, Jordan; i Department of Accounting, College of Business, Amman Arab University, Amman, Jordan; j Management Department, School of Business, King Faisal University, Kingdom of Saudi Arabia

**Keywords:** Saudi Arabia, Non-renewable energy, Renewable energy, Technological advancement, CO_2_ emissions, ARDL and VECM

## Abstract

This paper investigates the effects in short and long run of renewable and non-renewable energy, technological advancement, population, foreign direct investment, energy export, energy price, and carbon dioxide emissions on economic growth in Saudi Arabia as one of the largest oil producing and richest countries in the world and as a leading country in investing in modern technology, during 1990–2022 by using the Autoregressive Distributed Lag(ARDL) approach and the Vector Error Correction Model (VECM) Granger causality technique. In first step, the ADF and DF-GSL tests are used to identify the order of integration of variables. In the second step, the Bounds test and the Wald test are used respectively to verify the existence of long run cointegration relationships and the long run relationships between variables. In the third step, we have applied the ARDL approach to capture the effect of each variable on Saudi economic growth in long term. Finally, the VECM technique was used to detect the direction of causality running from variable to another. It is appearing that all variables are stationary in first difference, and there are a long run cointegration and relationships among variables. The results of ARDL estimation show that non-renewable energy, renewable energy, population, foreign direct investment, energy export, and energy price positively affect the Saudi economic growth. While technological advancement and carbon dioxide emissions have negative effects on the economic increase of Saudi Arabia. These two results appear important and useful because of their consequences. In effect, it could damage its worldwide standing and dishearten foreign investment, stopping economic diversification efforts and increasing the income inequality. Though, the results of VECM technique show four bidirectional causal relationships between economic growth and non-renewable energy, foreign direct investment, energy export, and energy price. The findings of this study have several policy implications for Saudi Arabia. First, Saudi government should continue investing in the energy sector. Second, to attract more FDI, Saudi government should continue its efforts to reduce bureaucracy, simplify regulations, and provide a business-friendly environment. This strategy can help transfer technology and knowledge. Third, the government should monitor and control energy prices, as these can significantly impact economic growth. The government should invest in technological advancement, as this can help reduce carbon dioxide emissions and improve energy efficiency; also, investing in human capital is essential for long-term economic growth. Policies that promote the health, education, and general well-being of the population can lead to a more productive and innovative workforce. However, the article reveals that technological advancements have a negative impact on economic growth in Saudi Arabia. This could be due to a number of factors, such as a lack of skilled workers to implement new technologies or a mismatch between the skills of the workforce and the needs of the economy. As solutions, Saudi government must invest in education and training can help address these challenges by developing a workforce capable of adapting to the changing needs of the economy and effectively using new technologies. Also, it's important to create science and technology parks to foster innovation and collaboration between businesses and universities. By taking these steps, the Saudi government can help create more diverse and knowledge-based economy, making it less dependent on oil and gas exports and more resilient to economic shocks.

## 1 Introduction

As a prominent global energy player, Saudi Arabia has historically relied on fossil fuels, particularly oil, to meet its energy needs. However, in recent years, the Kingdom has recognized the importance of diversifying its energy mix and promoting the adoption of renewable energy sources. This shift towards renewable energy is driven by various factors, including environmental concerns, energy security, and necessity to harness the Kingdom's abundant renewable energy resources. The dynamics of energy consumption and renewable energy adoption in Saudi Arabia are crucial areas of research, considering the country's significant energy demand and its commitment to reducing carbon emissions. Understanding the relationships between energy consumption, renewable energy adoption, and other relevant variables is essential for formulating effective policies and strategies to achieve a sustainable energy future. The Kingdom's commitment to renewable energy is reflected in its ambitious targets outlined in the Saudi Vision 2030 and the National Renewable Energy Program (NREP). The NREP aims to increase the share of renewable energy in the country's energy mix to 27% by 2030 m focusing on solar, wind, and nuclear energy [1].

Sustainable economic growth in Saudi Arabia is a difficult question. The Saudi economy depend on severely on oil and gas exports, making it susceptible to variations in global oil prices. The Saudi authority is knowledgeable of this brave and has taken actions to vary the economy, but lots stays to be resolved.

One of the main challenges to sustainable economic growth in Saudi Arabia is the need to reduce the country's dependence on oil and gas exports. The Saudi government has set a goal of reducing the country's dependence on oil to 50% by 2030, [1]. This is an ambitious goal, but it is achievable if the government continues to invest in non-profit sectors. oil industries such as tourism, manufacturing and technology.

Another challenge to sustainable economic growth in Saudi Arabia is the need to create jobs for the country's growing population. The Saudi population is expected to increase from 34 million today to 45 million by 2030, [2]. The Saudi government must create millions of new jobs to meet the needs of this growing population. This can be achieved by investing in education and training and creating a business-friendly environment that attracts foreign investment.

The Saudi government also faces the challenge of climate change. Climate change is already having a negative impact on the Saudi economy, and this impact is expected to worsen in the future. The Saudi government must invest in renewable energy and other clean technologies to reduce the country's greenhouse gas emissions and make the economy more resilient to climate change. Despite the challenges, there are a few opportunities for sustainable economic growth in Saudi Arabia. The country has a young and growing population, a well-educated workforce, and abundant natural resources. The Saudi government is also committed to diversifying the economy and investing in non-oil sectors.

The objective of this article is to explore the impact of energy consumption, technological progress, population, foreign direct investment, energy exports, energy price and carbon dioxide emissions of carbon on economic growth in Saudi Arabia between 1990 and 2022. This is an important topic to study because Saudi Arabia is a major oil producer, and its economy relies heavily on the oil and gas sector. However, the country also faces a few challenges, such as population growth, climate change and the need to diversify its economy.

However, further research is still needed to investigate the complex interactions between energy consumption, renewable energy deployment, economic growth, policy frameworks, and technological advancements. This research article aims to fill this research gap by utilizing the ARDL approach to analyze the dynamics of energy consumption and renewable energy adoption in the Kingdom of Saudi Arabia (KSA), providing valuable insights for policymakers, researchers, and industry stakeholders. Nevertheless, before the assessment for the impact of energy, technological advancement, population, foreign direct investment, energy export, energy price, and carbon dioxide emissions on economic growth, it is essential to define the constraints. Here are some examples of constraints that could be considered:

- Resource constraints: The availability of natural resources, such as oil and gas, can limit economic growth.
- Technological constraints: The development and adoption of new technologies may be limited by factors such as financing, skilled labor and intellectual property rights.
- Environmental constraints: Climate change and other environmental challenges can hamper economic growth.
- Social and political constraints: Social and political factors, such as war, corruption and inequality, can also hamper economic growth.

It is important to take these constraints into account when assessing the impact of the variables you mentioned on economic growth. For example, if some country faces severe resource constraints, it may be difficult to achieve high levels of economic growth, even if it invests heavily in renewable energy and other efficiency measures. energy.

However, this study is based on some following hypotheses, for which they must be verified. The first hypothesis is that renewable and non-renewable energies, population, technological advancement, energy export and energy price have positive impacts on Saudi economic growth. The second hypothesis is that CO2 emissions has a negative impact on Saudi economic growth. The third hypothesis is that Saudi GDP has bidirectional causal relationships with renewable and non-renewable energies, energy export, energy price and carbon dioxide. By applying both ARDL and VECM methods, our research discards radiance on the discriminated short and long-run effects of these elements on economic growth. This provides precious insights keen on how Saudi Arabia's economy strength develop in reply to diverse drifts and strategy interventions.

In effect, the unit root test was applied in order to detect the order of variables integration by using the Augmented Dickey Fuller (ADF) and Dickey Fuller with Generalized Least Squared (DF-GLS). The Wald test and the Bounds test are used respectively to determinate the long run relationships between variables and the long run cointegration between variables. The CUSUM and CUSUMSQ tests are used to verify the stability model during period time. Two estimations by using the ARDL approach are applied to determinate the effects of non-renewable energy, renewable energy, population, foreign direct investment, technological advancements, energy export, carbon emissions and energy prices on Saudi economic growth in short and in long term. The VECM was used to capture the direction causal relationships among them.

## 2 Literaturereview

### 2.1 Population and economic growth

Numerous studies have examined the patterns and drivers of energy consumption in Saudi Arabia, shedding light on the challenges and opportunities associated with the country's energy landscape. Alshehry and Belloumi [3] investigated the factors influencing energy demand in Saudi Arabia, highlighting the role of economic growth, population, urbanization, and energy prices. They found that economic growth and population were significant determinants of energy consumption, emphasizing the need for energy efficiency measures to mitigate the impact of rising energy demand. Meer et al. [4] have founded that population has positive impact on Pakistani economic growth in the short and long term. However, Fertility indicator negatively affect the economic growth in equally short and long term. Alshalalda [5] confirm that population element is the most important worry for many countries around the world. Which is particularly accelerated by industrial development and urbanization that generated some essential economic progression in many western countries.

However, Brahma [6] investigate the relationship between economic growth and the age structure of population. The results of empirical estimation show a significant negative relationship exists among these two variables, which suggests that, if nation has an increase in great amount of the dependent people, per habitat wages will verge to be decrease. Bawazir et al. [7], analytically investigates the impact of demographic adjustment on economic growth in Middle East region. The research discovers that indicators as population growth and employed-aged people positively stimulus growth. Nonetheless, the childhood addiction ratio negatively impacts economic growth. The research also notices that male labors provide additional certainly to economic growth than female labors and so additional attempts by the administration are necessary to participate females aggressively in the labor marketplace. In the same context, Temsumrit [8] prove that a rise in the old-age population meaningfully encourages advanced total government expenditure but only in advanced states and in specific on the expenditure in the social safety and environment types.

Bucci [9] shows that population growth and per capita individual capital growth are correlated to each other. In case of the might of this consequence go above failed positive level, population progress and per-capita wages progress can be adversely connected. This means that an adverse degree of population growing can withstand an optimistic economic growth degree in the long term. Wang et al. [10] confirm that human capital has a superior emission decrease consequence for the 208 countries studied subsequent to the EKC turning point.

### 2.2 R&D and economic growth

However, developing and deploying renewable energy technologies have become crucial in the global transition towards a sustainable energy future. Research and Development (R&D) expenditure is vital in driving innovation, improving efficiency, and reducing the costs of renewable energy technologies. This literature review provides insights into the relationship between renewable energy and R&D expenditure, as explored in existing studies. A growing body of research emphasizes the positive link between R&D expenditure and renewable energy development. Gallagher et al. [11] conducted a comprehensive study analyzing the impact of R&D investments on renewable energy innovation and deployment across countries. The study found that higher R&D spending was associated with increased renewable energy capacity, technology advancement, and cost reduction. The authors concluded that sustained R&D investments are essential for the continued growth and competitiveness of renewable energy industries.

Furthermore, studies have highlighted the significance of public and private sector R&D investments in promoting renewable energy innovation. Popp et al. [12] analyzed the effect of public R&D expenditure on renewable energy patents. The findings revealed a positive correlation between public R&D funding and patenting activity in renewable energy technologies, indicating the crucial role of government support in driving innovation. In addition to public investments, private sector R&D expenditure is a key driver of renewable energy innovation. Huenteler et al. [13] examined the relationship between private R&D investments and solar photovoltaic (PV) technology development. The study demonstrated that increased private sector R&D spending led to enhanced solar PV module efficiencies, lower production costs, and increased market competitiveness.

Moreover, studies have emphasized the importance of collaborative R&D efforts and knowledge-sharing networks in advancing renewable energy technologies. Böhringer et al. [14], examined the impact of international R&D collaborations on developing wind energy technologies. The research highlighted those collaborative R&D activities facilitated technology transfer knowledge diffusion and accelerated the adoption of wind energy across countries.

Wang et al. [15] investigate the impact of digital economy on economic growth in case of 97 countries. The estimation results show that digital economy has positive impact on CO2 emissions provided that natural source rent does not exceed the medium level.

The literature supports that R&D expenditure is vital in driving renewable energy innovation, technology advancement, and market competitiveness. Both public and private sector investments in R&D contribute to developing and deploying renewable energy technologies, enabling the transition towards more sustainable energy system. Continued and increased R&D expenditure in the renewable energy sector is essential for unlocking further advancements, cost reductions, and widespread adoption of renewable energy technologies.

### 2.3 FDI and economic growth

Research studies investigating the relationship between renewable energy consumption and Foreign Direct Investment (FDI) in the energy sector have yielded interesting findings. Numerous studies have found a positive correlation between renewable energy consumption and FDI in the energy sector. Higher levels of renewable energy consumption tend to attract greater FDI inflows. Authors such as Al-Mulali et al. [16], and Bhattacharya et al. [17] have contributed to this body of research. Research has shown that well-designed and supportive renewable energy policies are crucial in attracting FDI. Authors like Menyah and Wolde-Rufael [18], and Caetano et al. [19] have explored the impact of renewable energy policies on FDI inflows in the energy sector. FDI in the renewable energy sector often involves technology transfer, contributing to growth and development. Researchers such as Li et al. [20] have explored the role of technology transfer in the relationship between renewable energy consumption and FDI. However, the size and potential of country's renewable energy market are important factors influencing FDI. Countries with larger populations and higher energy demands tend to attract more FDI. Authors such as Tamazian and Rao, and Asiedu and Lien [21] have examined the relationship between market size and FDI in the renewable energy sector.

Appiah-Otoo et al. [22] prove that foreign direct investment has a significant influence on economic growth. The conclusion more show that the interface among renewable energy utilization and FDI has a significant influence on economic growth. Therefore, foreign direct investment advances renewable energy utilization to progressively effect economic increase in the West of Africa.

Using the ARDL approach, Tariq et al. and others [23,[23], [23], [24], [25], [26], [27]] confirm the existence of positive long run correlation between FDI, economic growth and renewable energy measured by the electricity consumption, nevertheless this correlation is negative in the short run.

However, Wang et al. [28] show that trade openness degree has asymmetry impacts on environment. In effect, trade openness directs to boost carbon emissions by the structure of industry. Though trade diversification directs to decrease carbon emissions. On another hand, Wang et al. [29] have analyzed the effect of trade openness and FDI on economic growth and carbon emissions in 114 nations. They have founded that FDI has a positive role at the same time on economic growth and CO2 emissions.

### 2.4 RNE, NREN and economic growth

Research studies have provided a valuable understanding of the relationships between renewable and nonrenewable energy consumption and economic growth. Nguyen and Le [30] discovered that nonrenewable energy consumption increases per capita earnings in the long term. Changes in Vietnam's per capita income growth is facilitated by changes in both nonrenewable and renewable energy use in the short term. However, previous shifts in nonrenewable energy usage have harmed Vietnamese people's present economic development. Ivanovski et al. [31] argue that nonrenewable energy consumption positively and substantially affects economic growth in OECD nations, with the coefficient function exhibiting an upward trend over time. The contribution of renewable energy consumption to economic growth in these countries is statistically insignificant for most of the period covered in this analysis.

Nonetheless, renewable and nonrenewable energy consumption contributes to non-OECD countries' economic growth. Awodumi and Adewuyi [32] demonstrate that in Nigeria, positive changes in nonrenewable energy consumption hinder growth but reduce emissions. Increased use of various energy sources in Gabon fosters economic development and improves the environment. In Egypt, the use of various energy sources promotes economic expansion while having little effect on environmental damage. Positive changes in Angola's nonrenewable energy usage help the country's economy thrive, while the impact on carbon emissions varies over time and depending on the energy source. Similarly, negative petroleum and natural gas consumption changes in Egypt and Nigeria have comparable influences. In Ghana, according to Gyimah et al. [33] there is a feedback effect between economic growth and renewable energy use, but there is no significant indirect influence of renewable energy consumption on economic growth.

Regarding renewable energy adoption, several studies have focused on the potential of solar and wind energy in Saudi Arabia. Khan et al. [34] conducted a comprehensive assessment of the solar energy potential in the Kingdom, highlighting its vast solar resources and the opportunities for large-scale solar power generation. They emphasized the importance of supportive policies, technology advancements, and international collaborations in driving the deployment of solar energy systems.

Furthermore, the literature has examined the policy landscape and government initiatives promoting renewable energy adoption in Saudi Arabia. Barhoumi et al. [35] analyzed the impact of government policies, including the National Renewable Energy Program (NREP), on renewable energy development. They concluded that the NREP has created a favorable environment for renewable energy projects, attracting domestic and international investments and contributing to the growth of renewable energy capacity in the Kingdom.

Several studies have also explored the challenges and barriers hindering the widespread adoption of renewable energy in Saudi Arabia. Li et al. [36] have demonstrated that economic growth leads to the increase of the environmental footprint, signifying that the EKC hypothesis is unacceptable. Natural resource rents reinforce the relation among economic growth and ecological footprint. By controlling corruption, it can be possible to crush the positive liaison among economic growth and environmental footprint. However, Wang et al. [10] show that renewable energy consumption can help to reduce dioxide carbon emissions for the 208 countries studied before the EKC turning point.

Nevertheless, renewable energy has significant total impact on economic growth. Therefore, an increase in renewable energy consumption has an overall positive effect on economic growth. Overall, the literature indicates a growing interest in understanding the dynamics of energy consumption and renewable energy adoption in Saudi Arabia.

## 3 Data, model specification and methodology

### 3.1 Data

The principal objective of this research is to analyze the relationship between economic growth (EG), non-renewable energy (NREN), renewable energy (REN), population (P), foreign direct investment (FDI), technological advancements (TA), energy export (EE), carbon emissions (CE) and energy prices (EP) in Arabie Saudi during 1990–2022 periods. The choice of this subject is based on the work of Sbia et al. [37] which addresses the impact of urbanization, financial development and energy consumption (measured by kwt of electricity consumption) on economic growth, case of United Arab Emirates (UAE) which is economically similar with the Arabia Saudi, during 1975–2011 periods, using the ARDL approach.

This study uses Gross Domestic Product per capita as proxy for economic growth (constant 2010 US$). Non-renewable energy was measured by kilograms of oil equivalent per capita. Renewable energy was measured by the solar power energy (number of giga watts). This indicator (solar power energy) is used to show the importance of solar energy in the Kingdom, as it is located within desert climate and is characterized by sunshine for long periods of the year. Population is used as an essential factor in the economic circuit. It reflects both source of labor (human capital) and source of consumption (internal consumption market) [38]. The price of one barrel of crude oil indicates the energy prices. The FDI was the Foreign direct investment (FDI) in the energy sector. The technological advancements were expressed by the research and development expenditure (% of GDP). The energy exports are used to describe that energy exports can be a source of government revenue and foreign investment and help reduce poverty levels. Finally, carbon dioxide (CO_2_) emissions (metric tons per capita)denote environmental degradation. The data on EG, NREN, REN, P, FDI, TA, EE, and CE shown in Table 1 have been gathered from the World Development Indicators online database (2023). However, the data on EP has been collected from OPEC (stands for the Organization of the Petroleum Exporting Countries), 2023.

**Table 1:** A summary of variables.

Variables	Meaning	Units	Sources
EG	**E**conomic **g**rowth	GDP per capita	WIDI, 2023
NREN	**N**on-**re**newable **en**ergy	kilograms of oil per capita	WIDI, 2023
REN	**R**enewable **en**ergy (solar power)	number of giga watts	WIDI, 2023
P	**P**opulation	millions of inhabitants	WIDI, 2023
FDI	**F**oreign **d**irect **i**nvestment(energy sector)	billion USD	WIDI, 2023
TA	**T**echnological **a**dvancements	% of GDP	WIDI, 2023
EE	**E**nergy **e**xport	billion USD	WIDI, 2023
CE	**C**arbon **e**missions	metric tons per capita	WIDI, 2023
EP	**E**nergy **p**rices	price of a barrel of crude oil	OPEC, 2023

This research paper investigates the relationship among EG, NREN, REN, P, FDI, TA, EE, CE, and EP in the case of the KSA economy with data spanning from 1990 to 2022.

Moreover, the descriptive statistics of our time-series data regarding the economy of the Kingdom of Saudi Arabia (KSA) are provided in Table 2. The findings indicate that all variables demonstrate a normal distribution, as indicated by the results of the Jarque-Bera test. An analysis of pair wise correlations reveals positive associations among the variables EG, NREN, RENE, P, FDI, TA, EE, CE, and EP.

**Table 2:** Descriptive statistics.

	EG	NREN	REN	P	FDI	TA	EE	CE	EP
*Average*	32,781	18,279	0.000	20.900	1.140	0.200	156.300	16.400	72.120
*Maximum*	87,516	33,910	0.300	34.800	8.900	0.600	204.400	28.900	147.270
*Minimum*	14,937	11,499	0.000	16.600	0.000	0.100	124.100	10.000	10.490
*Skewness*	0.480	0.420	3.090	1.420	1.920	0.710	2.060	1.180	2.230
*Kurtosis*	2.790	2.740	10.850	3.620	5.550	2.780	5.320	3.250	6.070
*Jarque-Bera*	1.560	1.430	43.220	3.720	23.380	1.940	7.520	2.480	27.330
*P-value*	0.000	0.000	0.000	0.000	0.000	0.000	0.000	0.000	0.000
Observations	33	33	33	33	33	33	33	33	33

The results in Table 2 indicate that the skewness of all variables in Saudi Arabia is slightly positive, indicating that the distribution is slightly right skewed. This shows more data points on the right side of the distribution. The kurtosis indicator is slightly higher than the normal distribution, indicating that the distribution is slightly leptokurtic. This means that the distribution has heavier tails than the normal distribution, which corresponds to more extreme levels of variables. The Jarque-Bera test is a statistical test that is used to test for normality. The p-value of the Jarque-Bera test in Saudi Arabia is 0.000, which is less than 0.05. This means that the distribution of variables in Saudi Arabia is normal.

### 3.2 Model specification

Our research paper investigates the factors and the nature of the relationships between EG, NREN, REN, P, FDI, TA, EE, CE, and EP within the Kingdom of Saudi Arabia (KSA) economy. Previous research conducted by Ifa and Guetat [39] has already examined the interplay among these key variables by using ARDL approach and VECM technique. The choice of the ARDL approach(developed by Pesaran et al. [40]) and the VECM technique has several advantages. In effect, the paper uses both the Autoregressive Distributed Lag (ARDL) approach and the Vector Error Correction Model (VECM) Granger causality technique. This combination of methods allows for in-depth analysis of short- and long-term relationships between variables, including causality tests. This methodological choice improves the robustness and depth of the analysis. The document takes into account a wide range of variables, including non-renewable energy, renewable energy, population, foreign direct investment, energy exports, energy price and carbon dioxide emissions. This comprehensive approach provides a comprehensive view of the factors that influence economic growth.

The ARDL approach can be used to estimate the long-term and short-term effects of variables on each other, even if the variables are integrated of a different order, I(0), I(1) or a mixture both. It's robust to endogeneity issues, meaning it can provide accurate estimates even if there is a bidirectional causal relationship between the variables. The ARDL approach is also robust to small sample sizes, [26,41]. However, the ARDL approach gives an opportunity to analyze how variables change over time and how they respond to shocks or policy changes. This dynamic analysis can provide valuable insights into Saudi Arabia's economic and energy dynamics over the years [42].

However, the VECM technique can be used to model long-term equilibrium relationships between variables, as well as the short-term dynamics of these relationships. It can also be used to identify causal relationships between variables. In effect, the VECM technique can be used to assess the impact of policy changes or external shocks on the system of variables. This is valuable for policymakers and researchers interested in understanding how various policy interventions affect the Saudi economy and energy sector. Finally, the VECM can be used to perform Granger causality tests (developed by Engle and Granger [43]), which help determine causal relationships between variables. This is essential to establish the direction of influence between economic growth, energy variables and other factors in Saudi Arabia.

In the specific case of our model, the ARDL approach and VECM technique can be used to estimate the long-term and short-term effects of non-renewable energy, renewable energy, population, foreign direct investment, progress technology, energy exports, carbon emissions and energy prices on Saudia economic growth. Also, it is necessary to test the presence of cointegration, which would indicate that there is a long-term equilibrium relationship between economic growth and the other explanatory variables. Finally, it is important to identify the causal relationships between economic growth and other exogenous variables.

To establish a connection between EG (economic growth), NREN (non-renewable energy), REN (renewable energy), P (population), FDI (foreign direct investment), TA (technological advancements), EE (energy export), CE (carbon emissions), and EP (energy prices), our study presents the following equation (1):

(1) EG=f(NREN,REN,P,FDI,TA,EE,CE,EP)

The model is defined as follows (equation (2)):

(2) $${EG}_{t}={\alpha_{0}{+\beta }_{1}NREN}_{t}+\beta_{2}{REN}_{t}{+\beta }_{3}P_{t}+\beta_{4}{FDI}_{t}+\beta_{5}{TA}_{t}{+\beta }_{6}{EE}_{t}+\beta_{7}{CE}_{t}+\beta_{8}{EP}_{t}+\varepsilon_{t}$$

Equation (3) as follow presents the natural logarithm of different variables from Equation (2):

(3) $${LnEG}_{t}={\alpha_{0}{+\beta }_{1}LnNREN}_{t}+\beta_{2}{LnREN}_{t}{+\beta }_{3}{LnP}_{t}+\beta_{4}{LnFDI}_{t}+\beta_{5}{LnTA}_{t}{+\beta }_{6}{LnEE}_{t}+\beta_{7}{LnCE}_{t}+\beta_{8\;\mathrm{Ln}}{EP}_{t}+\varepsilon_{t}$$

where:

$\alpha_{0}$ is the constant, $\beta_{1}$ to $\beta_{8}$ are the coefficients, $\varepsilon_{t}$ indicates the residual term, LnEG is the logarithm of EG, LnNREN is the logarithm of NREN, LnREN is the logarithm of REN, LnP is the logarithm ofP, LnFDIis the logarithm of FDI, LnTA is the logarithm of TA, LnEE is the logarithm of EE, LnCE is the logarithm of CE and finally LnEP is the logarithm of EP.

The expression of the ARDL model is represented by equation (4):

(4) $${DlnEG}_{t}=\beta_{0}+\sum_{i=1}^{p}\gamma_{i}{DlnEG}_{t˗i}+\beta_{1}{EG}_{t˗1}+\sum_{i=1}^{q}\delta_{i}{DlnNREN}_{t˗i}+\beta_{2}{NREN}_{t˗1}+\sum_{i=1}^{q}\epsilon_{i}{DlnREN}_{t˗i}+\beta_{3}{REN}_{t˗1}+\sum_{i=1}^{q}\vartheta_{i}{DlnP}_{t˗i}+{\beta_{4}P}_{t˗1}+\sum_{i=1}^{q}\mu_{i}{DlnFDI}_{t˗i}+\beta_{5}{FDI}_{t˗1}+\sum_{i=1}^{q}\pi_{i}{DlnTA}_{t˗i}+\beta_{6}{TA}_{t˗1}+\sum_{i=1}^{q}\tau_{i}Dln{EE}_{t˗i}+\beta_{7}{EE}_{t˗1}+\sum_{i=1}^{q}{ϧ}_{i}Dln{CE}_{t˗i}+\beta_{8}{CE}_{t˗1}+\sum_{i=1}^{q}{ϴ}_{i}Dln{EP}_{t˗i}+\beta_{9}{EP}_{t˗1}+\varepsilon_{t}$$

where:

The first-difference operator is represented by D. γ, δ, ϵ, θ, ϑ, μ, π, τ, ϧ and ϴ are error correction dynamics. $\beta_{1}$ to $\beta_{9}$ reveal the long-term relationships involving variables of ARDL model. The optimal lags are designed by p and q.

Before starting ARDL estimation, it is necessary to verify first of all the order of integration of all variables (check stationarity) using Augmented Dickey-Fuller (ADF) developed by Dickey and Fuller [44] generalized Least Squares (DF-GLS) unit root tests. Secondly, it's important to verify the existence or not of long-run cointegration between variables of model by using the Wald test. The F-statistic value should be less than 10% (according to Pesaran et al. [40]). The null hypothesis (H_0_) and the alternative hypothesis (H_1_) are as follow:

H0 $\beta_{1}=\beta_{2}=\beta_{3}=\beta_{4}=\beta_{5}=\beta_{6}=\beta_{7}=\beta_{8}=\beta_{9}=0$ (there are no long-term relationships between variables)

H1 $\beta_{1}\neq \beta_{2}\neq \beta_{3}\neq \beta_{4}\neq \beta_{5}\neq \beta_{6}\neq \beta_{7}\neq \beta_{8}\neq \beta_{9}\neq 0$ (there are long-term relationships between variables)

Thirdly, the Bounds test is used to detect the long run cointegration between variables of model.

In final step, the VECM technique (as restriction of VAR) is applied to capture the direction of short-term causality relationships among variables. However, The Error Correction Term (ECT) is the cointegration term that measures the speed at which endogenous variables converge to their long-run equilibrium. The coefficient of ECT should be significant and negative at the same time. It allows for short-term dynamic adjustments around the equilibrium.

Narayan and Smyth [45] have developed the VAR approach and it can be to write as follow in equation (5):

(5) $${DlnEG}_{t}=Z_{0}+\sum_{i=1}^{p}\gamma_{i}{DlnEG}_{t˗i}+\sum_{i=1}^{q}\delta_{i}{DlnNREN}_{t˗i}+\sum_{i=1}^{q}\epsilon_{i}{DlnREN}_{t˗i}+\sum_{i=1}^{q}\vartheta_{i}{DlnP}_{t˗i}+\sum_{i=1}^{q}\mu_{i}{DlnFDI}_{t˗i}+\sum_{i=1}^{q}\pi_{i}{DlnTA}_{t˗i}+\sum_{i=1}^{q}\tau_{i}Dln{EE}_{t˗i}+\sum_{i=1}^{q}{ϧ}_{i}{DlnCE}_{t˗i}+\sum_{i=1}^{q}{ϴ}_{i}Dln{EP}_{t˗i}+{Ɛ}_{t}$$

The VECM detects the long run relationship and the short run dynamics among the variables over the error correction term (ECT). In effect, the ECT determines the space between variables and their long run equilibrium. However, the VECM reveals how the variables in the econometric model adjust to their long run equilibrium over time.

The VECM model (equation (6)) is written as follow:

(6) $${DlnEG}_{t}={П}_{0}+\sum_{i=1}^{p}\gamma_{i}{DlnEG}_{t˗i}+\sum_{i=1}^{q}\delta_{i}{DlnNREN}_{t˗i}+\sum_{i=1}^{q}\epsilon_{i}{DlnREN}_{t˗i}+\sum_{i=1}^{q}\vartheta_{i}{DlnP}_{t˗i}+\sum_{i=1}^{q}\mu_{i}{DlnFDI}_{t˗i}+\sum_{i=1}^{q}\pi_{i}{DlnTA}_{t˗i}+\sum_{i=1}^{q}\tau_{i}Dln{EE}_{t˗i}+\sum_{i=1}^{q}{ϧ}_{i}{DlnCE}_{t˗i}+\sum_{i=1}^{q}{ϴ}_{i}Dln{EP}_{t˗i}+\varphi {ECT}_{t˗1}+{Ɛ}_{t}$$

In effect: П, γ, δ, ϵ, ϑ, μ, π, τ, ϧ, ϴ and φ are factors and ε is the white noise error term. ECT is error correction term.

## 4 Estimations and discussions

So, our empirical work goes through these steps or tests: ADF and DF-GLS tests, Wald test, Bounds test, ARDL estimation, and finally, Granger causality test.

### 4.1 Tests of unit root

The ADF and DF-GLS tests are used to detect the order of integration of variables (stationarity). The results indicated in Table 3 show that not all variables are stationary at level (I_0_), but in the first difference (I_1_), all variables are stationary, meaning that all variables are integrated in the first difference.

**Table 3:** Results of stationarity tests.

Tests	ADF test		DF – GLS test	
	Integration order		Integration order	
Variables	At level (I_0_)	First difference (I_1_)	At level (I_0_)	First difference (I_1_)
EG	−0.637	−6.310**	−1.213*	−5.663***
NREN	−3.083**	−8.003***	−3.088	−10.807***
REN	−4.711	−11.482***	−4.932**	−8.401***
P	−0.092	−4.700***	−0.042	−5.654***
FDI	1.474	−2.198*	1.758*	−2.593**
TA	0.920	−5.564***	0.983	−5.967***
EE	−0.258	−7.392***	−0.241	−6.007***
CE	2.641	−1.991*	1.097	−3.708**
EP	0.523	−3.372**	0.682	−5.228***

The significance at 1%, 5% and 10% thresholds are respectively indicated by*, ** and ***.

### 4.2 Test of wald

The Wald test is used to verify the existence of long run cointegration between variables. In effect, the F-statistic value (3.813) is significant at 1%, 5% and 10%, meaning that there is long-run cointegration between EG variable (dependent variable) and the rest of the variables (independent variables). The results are indicated in Table 4.

**Table 4:** Results of Wald test.

Test Statistic	Value	df	Prob.
F-statistic	3.813	(1, 29)	
Chi-square	3.813	1	0.004

The significance at 1%, 5%, and 10% thresholds are respectively indicated by *, ** and ***.

### 4.3 Test of bounds test

The Bounds test is used to verify the existence of long-run relationships among variables. The F-statistic value (10.812) of the Bounds test is significant at 1%, 5%, and 10% wish would mean that a long-run relationships between variables exist. The results are indicated in Table 5.

**Table 5:** Results of Bounds test.

F-statistic	Optimal lag length	F-statistic
**F**_**EG**_**(NREN, REN, P, FDI, TA, EE, CE, EP)**	(0,0,0,1,0,0,1,1,0)	10.812***
	**Critical Value bounds**	
Significance level	***I*(0)**	***I*(1)**
**10%**	1.74	2.96
**5%**	2.17	3.31
**1%**	2.65	3.97

The significance at 1%, 5%, and 10% thresholds are respectively indicated by *, ** and ***.

### 4.4 Stability test

**Fig. 1:**
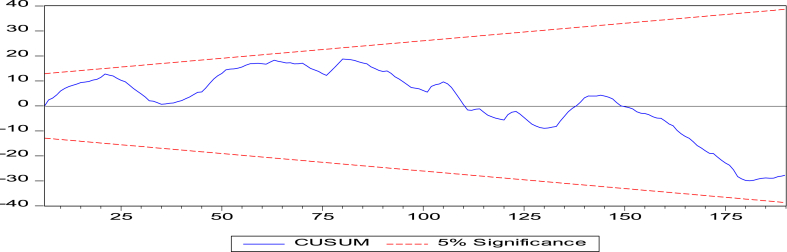
CUSUM test.

**Fig. 2:**
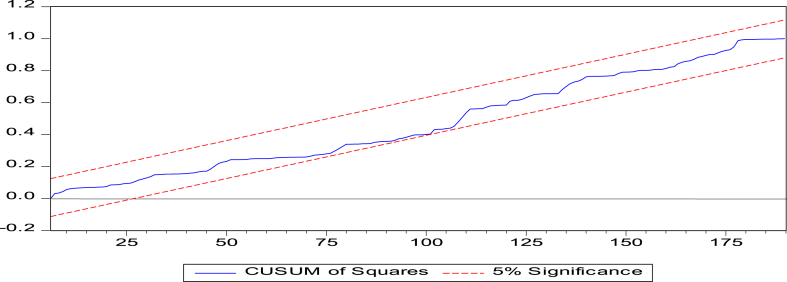
CUSUMSQ test.

Note: The CUSUM and CUSUMSQ axes are statistical control tools (their scales are chosen automatically by the Eviews 10 software) used to detect changes in the mean of a variable over time. They are based on the notion of cumulative sum (cusum), which is the cumulative sum of the differences between the observed value of a variable and its mean.

The CUSUM and CUSUMSQ tests are used to test the stability of our model. The results in Table 6 show that the econometric model was stable during the estimation periods 1990–2022. As shown in Fig. 1, Fig. 2, the model curve fluctuation (colored in blue) was inside the ranges (colored in red).

**Table 6:** Results of long run ARDL estimations.

Independent variables	Coefficients	Std. Error	t-Statistic	Probabilities
LnNREN	0.573	0.067	8.468	0.000***
LnREN	0.010	0.003	2.767	0.016**
LnP	0.007	0.003	2.008	0.046**
LnFDI	0.539	0.058	9.262	0.083*
LnTA	−0.009	0.033	−0.286	0.775
LnEE	4.891	0.279	17.478	0.094*
LnCE	−0.073	0.052	−1.412	0.159
LnEP	3.762	0.341	11.009	0.000***
Constant	0.017	0.047	0.376	0.707

Stability tests	CUSUM test	Stable
	CUSUMSQ test	Stable

The significance at 1%, 5% and 10% thresholds are respectively indicated by *, ** and ***.

### 4.5 Long run ARDL estimation

The results of ARDL estimation show that six variables (NREN, REN, P, FDI, EE and EP) among eight variables have positive effects on Saudi economic growth. The results show that an increase of NREN by one-unit positively and significatively affects EG by 0.573% Mahalik et al. [46] confirm this positive effect. Economically, this phenomenon can be attributed to combination of factors that underscore the economic significance of non-renewable energy sources in the Saudi context. Firstly, Saudi Arabia possesses vast reserves of non-renewable energy resources, particularly oil. These resources have endowed the nation with unique comparative advantage in global energy markets, enabling it to tap into substantial revenue streams through exports.

As major oil exporter, the country has been able to leverage its energy reserves to generate significant foreign exchange earnings, bolstering its balance of payments and providing a substantial source of government revenue. Secondly, exploiting and producing non-renewable energy resources have driven extensive downstream industries within Saudi Arabia. The petrochemical sector, for in-stance, has grown in tandem with the energy industry, utilizing oil-derived feed stocks to manufacture diverse array of products, from plastics to fertilizers. This diversification into value-added industries has generated employment opportunities, stimulated innovation, and enhanced economic resilience, thereby contributing to the nation's overall economic growth. Furthermore, the revenue generated from non-renewable energy exports has enabled Saudi Arabia to undertake ambitious infrastructure projects and invest in various sectors of the economy. This has facilitated economic diversification initiatives, as the government has channeled these funds into sectors such as finance, tourism, and technology, seeking to reduce the economy's reliance on oil and create more sustainable growth trajectory.

Nevertheless, while the positive role of non-renewable energies in Saudi economic growth is evident, it's essential to recognize the long-term challenges associated with their use. In conclusion, the positive economic impact of non-renewable energies on Saudi Arabia's growth trajectory underscores their pivotal role in shaping the nation's economic landscape, even as the imperative for sustainable energy practices looms ever larger.

An increase of REN by one-unit positively and significatively effects EG by 0.01% Namahoro et al. [47] have founded the same result. The Saudi population variable (P) has a slight positive and significative effect of 0.007% on EG; the same result was proved by Dao [24]. However, renewable energies play pivotal role in driving Saudi economic growth due to their positive economic externalities. By reducing reliance on finite fossil fuels, these sustainable energy sources enhance energy security, foster innovation, and create new economic opportunities in emerging industries. This transition promotes long-term economic resilience by mitigating environmental risks and ensuring diversified energy portfolio, ultimately contributing to sustained economic expansion.

The FDI leads to increase EG by 0.539% Jiang, Y and Martek [48]. In effect, Foreign Direct In-vestment (FDI) plays a crucial role in driving Saudi Arabia's economic growth by infusing external capital, expertise, and technology into the domestic economy. The influx of foreign capital provides access to funds that can be allocated towards productive sectors, stimulating job creation and boosting economic activity. Additionally, FDI facilitates knowledge and technology transfer, fostering innovation and efficiency improvements within local industries, collectively contributing to sustained economic expansion.

However, the energy export (EE) and the energy price (EP) variables have significant effects on increasing Saudi economic growth. An increase of EE according to Ali and Hameed [49], and of EP Shen et al. [50] by 1% serve to increase the EG variable respectively by 4.891% and by 3.762%. In economic term, energy export (EE) has a crucial positive role in Saudi economic growth by tapping into global demand for energy resources. As one of the world's largest oil exporters, Saudi Arabia benefits from the substantial revenue generated through energy exports, bolstering its trade balance and national income. Simultaneously, the energy price (EP) serves as a catalyst, as higher energy prices enhance the value of exports, magnifying the economic gains from energy trade and contributing to the expansion of the national economy.

However, Saudi economic growth was negatively affected by the increases of TA [51] and CE [52] variables. In effect, the expansion of TA result contradict what Wang and Wu [53], and an increase of CE by 1% led to decrease Saudi economic growth respectively by 0.009% and by 0.073%; (result contradict what Halkos and Gkampoura [54] have found. The negative impact of technological advancement and carbon emissions on Saudi economic growth can be attributed to distinct economic dynamics. This is an original finding evaluated to existing studies, which frequently centers primarily on the positive features of technological advancements and the economic costs of climate change in broader sense. Technological advancement, while essential for long-term productivity gains, might initially lead to resource reallocation and skill mismatch, impeding short-term growth. Similarly, elevated carbon emissions, indicative of inefficient resource use, can result in environmental degradation, prompting regulatory constraints and resource scarcity that curtail economic output in long run.

### 4.6 Short run causality and ECT test

The results of ECT in Table 7 show that there is at least one long term relationship among exogenous variables and EG as dependent variable (the coefficient should be negative and significative). It means that EG variable has an essential part as alteration component once the econometric model diverges from equilibrium.

**Table 7:** Granger causality results and ECT test.

Causality method										
Short run										Long-run
	**DLnEG**	**DLnNREN**	**DLnREN**	**DLnP**	**DLnFDI**	**DLnTA**	**DLnEE**	**DLnCE**	**DLnEP**	**ECT**
DLnEG	–	0.325** (0.034)	4.002 (0.932)	0.931 (0.342)	7.881*** (0.004)	3.039 (0.219)	0.762* (0.057)	1.432* (0.093)	0.022*** (0.007)	−2.092* (0.058)
DLnNREN	2.892** (0.047)	–	0.032 (0.642)	1.098 (0.376)	3.039** (0.022)	0.873 (0.727)	4.993*** (0.003)	2.083** (0.021)	2.172*** (0.008)	−0.114** (0.041)
DLnREN	0.013 (0.945)	−0.020 (0.349)	–	0.038 (0.782)	−0.287 (0.820)	1.802* (0.052)	−0.542 (0.839)	−1.902 (0.896)	−0.064 (0.792)	0.982 (0.829)
DLnP	0.067 (0.849)	1.048** (0.044)	−0.003 (0.947)	–	0.204 (0.911)	0.008 (0.732)	1.960 (0.783)	2.528* (0.058)	0.962 (0.706)	1.673 (0.804)
DLnFDI	3.603* (0.086)	3.902 (0.060)	0.965 (0.563)	0.022 (0.892)	–	2.962 (0.091)	1.841 (0.121)	0.987 (0.552)	0.471** (0.023)	0.829 (0.562)
DLnTA	−0.015 (0.603)	−0.027 (0.639)	1.938** (0.038)	0.036 (0.826)	−0.055 (0.120)	–	0.067 (0.782)	0.830 (0.928)	0.620 (0.830)	0.762 (0.852)
DLnEE	5.060*** (0.009)	2.803*** (0.003)	0.036 (0.187)	0.904 (0.402)	4.003 (0.632)	0.039 (0.506)	–	6.034* (0.060)	1.728** (0.039)	−1.991** (0.055)
DLnCE	0.933 (0.180)	0.837 (0.431)	−0.079 (0.475)	0.668 (0.103)	0.372 (0.601)	0.932 (0.582)	0.802 (0.932)	–	4.626 (0.852)	0.841 (0.569)
DLnEP	1.830** (0.011)	2.993** (0.048)	0.051 (0.681)	0.602 (0.669)	0.793** (0.028)	0.261 (0.891)	0.553 (0.872)	0.036* (0.082)	–	−0.941*** (0.008)

The significance at 1%, 5% and 10% thresholds are respectively indicated by *, ** and ***.

However, the Granger causality test shows mixed causal relationships between variables (unidirectional and bidirectional).

The short-run results indicate that our dependent variable EG (Economic Growth) has four bidirectional causal relationships with NREN, FDI, EE, and EP. Started by explaining the relationship between EG and NREN, so it appears that fossil energies (NREN) have an important role in Saudi economy. In effect, the increase of fossil production energies or the new explorations and exploitations of energy fields give positive reviews on Saudi economy, which will encourage Saudi and foreign investors to invest in this Kingdom. However, the increases of EG provides financial and technical capabilities, part of which will be used for investment in energy sector.

The FDI variable positively causes EG. In economic terms, FDI has advantages in economic growth like importing technologies, technical competencies, and experiences in energy field. Conversely, the increase in economic growth is considered an important factor for attracting FDI.

The Saudi economy is based on energy export, which means that the increase of EE has a positive effect on EG in Saudi Arabia, and the expansion of EG allows investment in infrastructure, ports, oil tankers and supply lines. However, the increase of EP represents a prospect for Saudi economy to achieve additional gains and financial resources. Nevertheless, strong economy allows the Saudi government to control energy production and thus control global energy prices, as it is an essential member of OPEC (Organization of the Petroleum Exporting Countries).

The short-run results also show that NREN has one more bidirectional causal relationship with EE and two unidirectional causal relationships with P and EP (P and EP cause EG). A bidirectional causal relationship exists between TA and REN. The FDI has a bidirectional causal relationship with EP variable and an unidirectional causal relationship with NREN (NREN causes FDI). Finally, an unidirectional causal relationship exists from EE variable to EP variable.

**Fig. 3:**
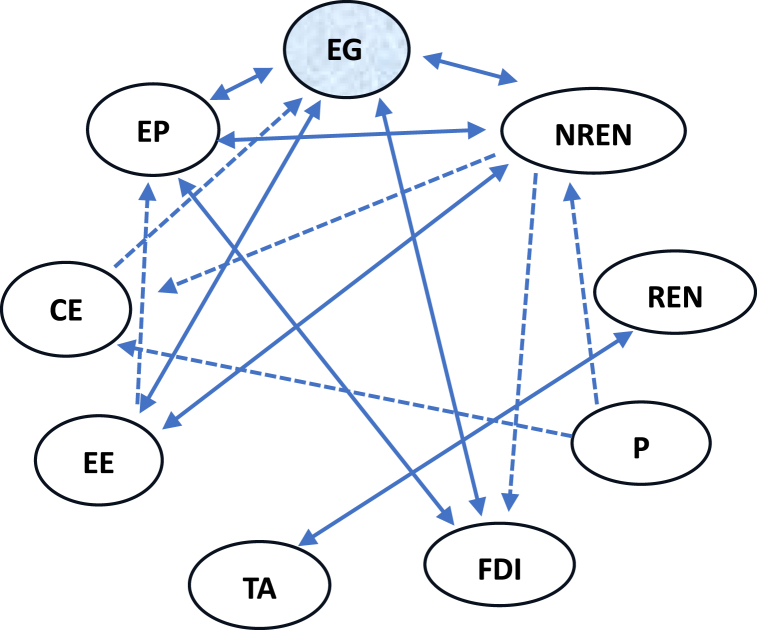
Directions of causal relationships. Note: (←→) indicates a bidirectional causal relationship. (→) indicates a unidirectional causal relationship.

In effect, Fig. 3 recapitulates all these causal relationships. It's appearing that EG variable (endogenous variable) has 4 bidirectional causal relationships with EP, EE, FDI and NREN. Only CE variable has an unidirectional causal relationship on EG variable.

## 5 Conclusion

In conclusion, this study examined the intricate relationships between various economic and environmental factors and their impact on Saudi Arabia's economic growth during the period of 1990–2022. Significant insights have been garnered through the application of ARDL approach and VECM technique. The analysis demonstrated that NREN, REN, P, FDI, EE, EP all contribute positively to Saudi economic growth. These findings emphasize the strategic importance of energy trade and pricing policies for Saudi economy.

However, the study also identified two factors with adverse effects on Saudi economic growth. In effect, TA and CE variables exhibited negative impacts on Saudi GDP rate. These results underscore the need for innovative policies to mitigate technological inefficiencies and environmental impacts. According to this result, it is particularly noteworthy that TA negatively affects GDP, because it contradicts the results of most other studies. In sum, the study contributes by highlighting the complex relationship between various factors and economic growth in the Kingdom of Saudi Arabia. The findings that technological advancements and CO2 emissions have negative impacts on economic growth are particularly noteworthy. This confront the conservative knowledge that technology is forever advantageous and highlights the probable pitfalls of quick technological transform, particularly in resource-needy economies as Saudi Arabia. The VECM analysis enlightening four bidirectional causal relationships amid economic growth and non-renewable energy, FDI, energy exports, and energy price is an additional significant contribution. This gives a deeper considerate of composite feedback loops and dynamic connections inside Saudi economy.

Considering these findings, several key policy implications emerge. The Saudi government should continue prioritizing investments in energy sector, encouraging both non-renewable and renewable sources while capitalizing on the benefits of FDI by creating an attractive business environment. The promotion of energy exports and careful control of energy prices can be leveraged to bolster revenue generation. To counter the adverse effects of technological advancement and carbon dioxide emissions, targeted initiatives aimed at enhancing technological efficiency and reducing environmental impact should be pursued.

Furthermore, improvements in infrastructure, particularly transportation networks, can amplify the effectiveness of these strategies by facilitating movement of goods and services. This study furnishes valuable insights for policymakers, providing a comprehensive understanding of factors shaping Saudi Arabia's economic growth and pointing toward informed, holistic strategies for sustainable development in years ahead.

However, The study allows some of limitations, like the use of a relatively short period (33 years). The empirical part does not tab for all elements that could potentially affect the economic growth in Saudi Arabia, and it does not describe for the potential endogeneity of some of variables. In addition, this article suggests a number of directions for future research, including the conduction a similar study over a longer period of time. Also, it is important to include additional variables in the analysis, such as the quality of governance and the level of corruption and it is necessary to use more sophisticated econometric techniques to address potential endogeneity of some variables.

### 5.1 Policy implications

This study gives as an opportunity to identify some positive and negative points. As positive strategy, Saudi government should sustain for energy diversification. In effect, the positive effect of RNE and FDI things to see the require for sustained investments in RNE and efforts to draw foreign resources. This preserve vary the economy, decrease dependence on NREN, and draw information and technology transfer. Moreover, it is important to organize energy resources by finding on energy exports and prices highlight the significance of planned organization of these resources. In addition, it is very important to manage energy prices and to look for new export targets, which can steady income and alleviate economic instabilities.

However, it is important to address technological commotions. In effect, the negative effect of TA proposes a require for practical plans. It is appearing that investing in education and preparation and generating R&D can provide the labor force to become accustomed to and influence new technologies efficiently. Moreover, the harmful effect of CO2 emissions requires direct act. It is important to look for clean energy solutions, carbon imprison and storeroom technology, and sustainable progress practices can generate a greener economy and preserve environment.

### 5.2 Limitations and future recommendations

Though this research gives precious approaches interested in drivers of Saudi Arabia's economic growth, it has a few limitations and proposes rich opportunities for future studies. We can give some limitations concerned the data scope, model limitations and unobserved factors. In effect, meeting point only on 1990–2022 strength ignore past trends and budding dynamics. Intensifying the study time could offer an additional comprehensive representation. However, the ARDL and VECM techniques, even as practical, present precise assumptions and probable limitations. Discovering balancing econometric methods can put in robustness and shade. Finally, external factors as worldwide economic trends or political instability in some region in the word strength not be completely detected in the model.

As future recommendations, the investigation in the “black box” of technological impact can disentanglement the precise devices why TA negatively affect growth of Saudi Arabia. It needs additional investigate. Is it ability disparity, dislocation of employees, or lack of infrastructure. Also, it is important to discover how growth affects diverse profits groups and sectors.

## Data availability

Data will be made available on request.

## CRediT authorship contribution statement

**Faten Derouez:** Investigation, Formal analysis, Data curation, Conceptualization. **Adel Ifa:** Software, Resources, Methodology. **Abdullah A. Aljughaiman:** Validation, Methodology, Formal analysis, Data curation. **Mohammed Bu Haya:** Software, Methodology, Funding acquisition. **Abdalwali Lutfi:** Writing – original draft, Investigation, Formal analysis. **Mahmaod Alrawad:** Writing – review & editing, Supervision, Resources, Project administration, Funding acquisition. **Samah Bayomei:** Writing – review & editing.

## Declaration of competing interest

The authors declare that they have no known competing financial interests or personal relationships that could have appeared to influence the work reported in this paper.
